# Segmental Non-Mass Enhancement Features in Breast Magnetic Resonance Imaging: A Multicenter Retrospective Study of Histopathologic Correlations

**DOI:** 10.3390/diagnostics15233084

**Published:** 2025-12-04

**Authors:** Hale Aydin, Cansu Bozkurt, Serhat Hayme, Almila Coskun Bilge, Pelin Seher Oztekin, Aydan Avdan Aslan, Irem Ozcan, Serap Gultekin, Abdulkadir Eren, Irmak Durur Subası

**Affiliations:** 1 Department of Radiology, Ankara Gulhane Training and Research Hospital, University of Health Sciences, 06010 Ankara, Turkey; cansubozkurt.3087@gmail.com (C.B.); driremozcan@gmail.com (I.O.); 2 Department of Biostatistics and Health Informatics, School of Medicine, Erzincan Binali Yildirim University (EBYU), 24100 Erzincan, Turkey; serhathayme@gmail.com; 3 Department of Radiology, Ankara Dr. Abdurrahman Yurtaslan Oncology Training and Research Hospital, University of Health Sciences, 06200 Ankara, Turkey; almilacoskun@gmail.com; 4 Department of Radiology, Ankara Training and Research Hospital, University of Health Sciences, 06230 Ankara, Turkey; pelinoztekintr@yahoo.com; 5 Department of Radiology, Gazi University Hospital, 06560 Ankara, Turkey; aydanavdanaslan@gmail.com (A.A.A.); sergultekin@yahoo.com (S.G.); 6 Department of Radiology, Istanbul Medipol University Hospital, 34214 Istanbul, Turkey; aeren@medipol.edu.tr; 7 Department of Senology, Senology Institute, Acibadem Atakent Hospital, Acibadem Mehmet Ali Aydinlar University, 34303 Istanbul, Turkey; irmakdurur@yahoo.com

**Keywords:** multiparametric breast magnetic resonance imaging, segmental nonmass enhancement, breast cancer, clustered ring enhancement, contrast kinetics

## Abstract

**Background/Objectives**: Segmental non-mass enhancement (NME) is the breast MRI distribution pattern with the highest positive predictive value (PPV) for malignancy. Despite its diagnostic relevance, its imaging characteristics have rarely been examined in isolation, leaving uncertainty in clinical practice. This multicenter retrospective cohort study aimed to evaluate multiparametric MRI features—including internal enhancement pattern, dynamic contrast-enhanced (DCE) kinetics, and diffusion restriction—in segmental NME to identify malignancy predictors. **Methods**: This retrospective cohort review included 14,834 breast MRI reports from five institutions (September 2017–February 2024), identifying 103 women (mean age, 44.4 ± 9.9 years) with segmental NME (70 malignant, 33 benign). MRI was performed at 1.5 T or 3 T using standardized protocols. Two breast radiologists, blinded to pathology, assessed internal enhancement, DCE kinetics, diffusion restriction, and short tau inversion recovery (STIR) features according to BI-RADS. Statistical analyses included chi-square/Fisher’s tests and logistic regression. **Results**: Clustered ring enhancement (CRE) was significantly associated with malignancy (*p* = 0.004). Fast initial-phase enhancement (*p* < 0.001) and delayed-phase washout (*p* = 0.011) also correlated with malignancy. On multivariate analysis, fast initial-phase enhancement remained an independent predictor (odds ratio [OR] = 5.133, *p* = 0.031), whereas slow enhancement predicted benignity (OR = 0.194, *p* = 0.020). Histologies included ductal carcinoma in situ, invasive ductal carcinoma, granulomatous mastitis, and benign hyperplastic lesions. **Conclusions**: This study, focusing exclusively on segmental NME, identifies CRE, fast initial-phase enhancement, and washout kinetics as reliable imaging biomarkers. Incorporating these features into breast MRI interpretation may improve diagnostic accuracy, risk stratification, and management decisions.

## 1. Introduction

According to the Breast Imaging Reporting and Data System (BI-RADS) lexicon of the American College of Radiology (ACR), nonmass enhancement (NME) is defined as an enhanced lesion without a space-occupying, three-dimensional, convex-edged mass on dynamic contrast-enhanced (DCE) MRI of the breast. NME lesions do not cause distortion or displacement in the surrounding tissues, and islands of fatty tissue can be observed within them. In the ACR BI-RADS Atlas 5th edition, nonmass areas of contrast enhancement are classified according to their distribution characteristics and internal contrast enhancement patterns (IEPs) [1]. Histopathological diagnostic results for NME lesions can be classified as benign, high-risk, or malignant. Among the malignant lesions presenting as NME, ductal carcinoma in situ (DCIS) is the most common subtype; however, a substantial proportion also comprises invasive carcinomas, in agreement with recent meta-analytic and multicenter data [2,3,4]. In the study by Lui et al., 57.1% of malignant NME lesions were diagnosed as DCIS, and 42.9% as invasive carcinomas [3]. This finding emphasizes the need for cautious assessment of invasive potential.

In the literature, NME is commonly assessed using distribution and IEP descriptors, together with kinetic (time-intensity) curves and diffusion-weighted imaging (DWI)/apparent diffusion coefficient (ADC) values [1,4,5,6,7]. Distribution pattern and IEP play important roles in assessing the risk of malignancy. Segmental distribution (a wedge/cone-shaped enhancement following a single ductal system) combined with clustered ring enhancement (CRE; numerous small ring-like foci forming a clustered internal enhancement pattern) has the most significant correlation with malignancy, as demonstrated by a descriptor-level meta-analysis [1,3,5,8].

CRE with segmental distribution improves the positive predictive value (PPV) for detecting DCIS, and both features are widely accepted as important radiological markers [9]. Alongside distribution and IEP, diffusion restriction, signal characteristics on short tau inversion-recovery (STIR) sequences, and time–intensity curves (TIC) are evaluated to inform benign–malignant differentiation of NME lesions [4,8,10,11,12,13,14,15,16]. Several studies have demonstrated that adding DWI/ADC metrics to DCE-based assessment can increase specificity to some extent and aid in the characterization of NME lesions; however, this incremental benefit is limited in certain cohorts, particularly for lesions without a corresponding mass [6,14,16]. STIR- and T2-based sequences can facilitate discrimination of true NME from background parenchymal enhancement and post-treatment changes by depicting edema and perilesional alterations; nevertheless, they are not sufficient on their own for reliable benign–malignant differentiation [10,15]. A recent meta-analysis demonstrated that DCE combined with DWI yields the highest diagnostic accuracy (AUC ≈ 0.90), DCE alone the lowest, and that segmental/TIC-based assessments outperform IEP-based approaches; nevertheless, no single modality or descriptor can definitively confirm or exclude malignancy [6]. Notably, inter-study heterogeneity driven by protocol and reader variability remains substantial [7]. These uncertainties emphasize the need for further multiparametric analysis in well-characterized lesion subgroups. Although previous studies have generally evaluated NME lesions as a single group, their imaging features have rarely been analyzed in detail based on their distribution patterns [2,3,4]. This lack of stratification creates ambiguity in clinical decision-making, particularly when assessing malignancy risk based on distribution or internal enhancement patterns. To address this gap, our study focused specifically on segmental NME, the distribution pattern with the highest reported PPV for malignancy, to investigate associated multiparametric MRI characteristics in detail.

Distinguishing NME lesions from the background parenchymal enhancement can be challenging. The background parenchymal enhancement commonly exhibits mild, persistent kinetic curves and bilateral symmetrical distribution. However, in some cases, asymmetrical patterns may mimic NME, which complicates differentiation. Baltzer et al. reported that the diagnostic performance of breast MRI in NME evaluation is highly dependent on the reader experience [15]. However, despite multiple studies on NME, large multicenter cohorts—particularly those stratified by distribution pattern—remain limited. This underscores the need for larger, standardized series and distribution-based stratification.

We hypothesized that CRE and specific early-phase kinetic curves would be the most predictive multiparametric MRI features for malignancy in segmental NME lesions. Therefore, the primary aim of this multicenter, retrospective cohort study was to identify which MRI features—including internal enhancement patterns, DCE kinetics, DWI/ADC values, and T2-weighted/STIR characteristics—are most strongly associated with malignancy. A secondary aim was to determine the ability of these features to differentiate invasive carcinoma from DCIS within segmental NME, thereby highlighting the current limitations of imaging for predicting invasion.

Our study focuses specifically on the segmental NME subtype, which has the highest reported PPV for malignancy, unlike prior studies that often pool all NME patterns. This stratified approach is designed to provide lesion-specific, multiparametric MRI markers for segmental NME, addressing a key evidence gap and improving diagnostic precision and clinical decision-making for this finding.

## 2. Materials and Methods

### 2.1. Study Design and Population

**Figure 1:**
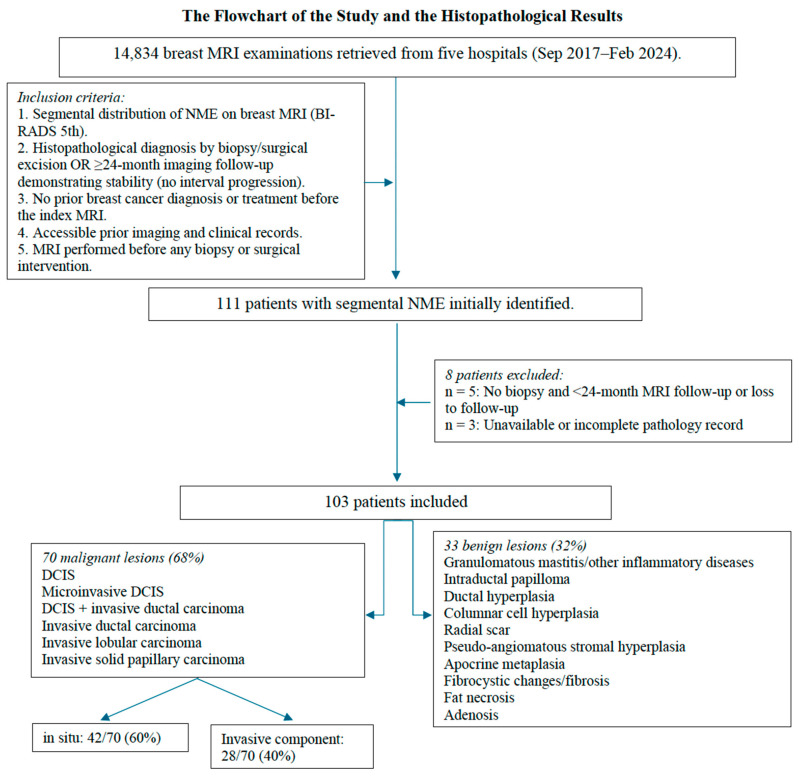
Flowchart of the study and histopathological results.

This multicenter retrospective cohort study was approved by the institutional ethics committee (Approval No. 2024-06/81; dated 11 July 2024). Informed consent was waived owing to the retrospective design and the use of anonymized, pre-existing MRI and histopathology data. We reviewed breast MRI and pathology reports acquired between September 2017 and February 2024 across five hospitals. Among 14,834 consecutive breast MRI examinations performed for clinical indications during the study period, 111 patients with segmental NME were initially identified. To minimize selection bias, all potentially eligible cases were identified in a consecutive fashion based on the predefined imaging and histopathologic criteria. All adult women undergoing clinical breast MRI during the study period were eligible, and no additional age-based inclusion or exclusion criteria were applied, because segmental NME lesions can occur across a wide adult age range. In the final cohort, patient age ranged from 22 to 75 years (mean ± SD, 44.4 ± 10.0 years). The inclusion criteria were as follows: (1) NME lesions with segmental distribution on breast MRI, (2) histopathological diagnosis established by biopsy/surgical excision or ≥24-month MRI follow-up demonstrating stability (no interval progression or BI-RADS upgrade), (3) no prior history of breast cancer diagnosis or treatment before MRI, and (4) accessible prior imaging and clinical records, (5) MRI performed prior to any biopsy or surgical intervention. The exclusion criteria were: (1) unavailable or incomplete pathology records; (2) no biopsy and <24-month MRI follow-up, or loss to follow-up; (3) MRI follow-up images that could not be retrieved; (4) MRI without essential sequences or with non-diagnostic image quality. Eight patients with incomplete pathology data or a lack of adequate follow-up were excluded; 103 patients were finally included. Surgical resection was performed in all malignant cases, whereas benign lesions were excised or followed up according to histopathologic risk. Figure 1 depicts the inclusion and exclusion process.

The biopsy and follow-up results were recorded. Lesions were confirmed via targeted ultrasonography in 66 patients, followed by US-guided core biopsies. MRI-guided biopsies were performed in three patients with lesions detectable exclusively on MRI, while MRI–ultrasound (MRI–US) fusion-guided biopsies were conducted in an additional three patients. Three patients underwent excisional biopsy under mammography guidance. Patients who did not undergo immediate biopsy received MRI follow-up for ≥24 months (6–12-month intervals); radiologic stability—no interval progression in extent and no BI-RADS upgrade—was considered benign, whereas progression prompted biopsy. The surgical pathology results were included for patients with malignancy diagnosed via core biopsy who received neoadjuvant chemotherapy. The histopathological diagnoses were classified as benign or malignant, and whether malignant NME lesions were invasive or in situ was recorded.

### 2.2. MRI Acquisition Protocol and Image Analysis

MRI scans were retrospectively reviewed between March and June 2024 by two breast radiologists (with 7 and 24 years of experience) who were blinded to histopathologic results. Two breast radiologists independently evaluated all studies; disagreements were resolved by consensus. The breast MRI protocol included axial seT1, fat-suppressed T2, diffusion-weighted imaging (DWI) (b = 0 and b = 800), and at least five-phase DCE sequences. Examinations without any of these sequences or with significant artifacts were excluded from the analysis according to predefined quality criteria in order to avoid misclassification due to suboptimal image quality. Examinations were performed at five hospitals on 1.5 T and 3 T scanners from GE Healthcare (Chicago, IL, USA) and Siemens Healthineers (Erlangen, Germany) across the participating centers.. Although TR/TE and matrix settings varied slightly across centers, the overall protocol structure was comparable. DCE-MRI included at least five post-contrast phases, with a temporal resolution of approximately 60–90 s per phase (total dynamic acquisition time about 6–8 min). DWI was acquired with b values of 0 and 800 s/mm^2^ at all sites; therefore, there was no inter-center variation in b-value selection.

Internal enhancement patterns (IEPs) were evaluated in all lesions according to the ACR BI-RADS 5th edition. If more than one IEP was present within a lesion, the descriptor with the highest positive predictive value (PPV) for malignancy was assigned as the primary IEP in the following order: CRE, clumped, heterogeneous, and homogeneous [2]. In addition, when two or more IEP descriptors coexisted within the same segmental NME lesion (e.g., CRE with clumped or heterogeneous components), this was recorded as a mixed enhancement pattern. Assessment of mixed IEP was feasible in 75 of the 103 lesions, in which it was analyzed as a separate binary variable (present/absent). In the remaining 28 lesions, internal enhancement could be reliably categorized by a single dominant IEP only, and mixed IEP was therefore not evaluated as a separate descriptor.

Diffusion restriction was considered present when the ADC value was below 1000 × 10^−6^ mm^2^/s, consistent with prior studies reporting ADC cut-offs around 1.0 × 10^−3^ mm^2^/s for differentiating malignant from benign breast lesions, particularly in NME cohorts [8,17]. The presence of cystic structures (focal high-signal foci interpreted as microcysts) on fat-suppressed T2-weighted or STIR sequences was recorded. Diffuse or non-cystic T2/STIR hyperintensity was not evaluated as a separate variable. Initial-phase contrast enhancement was categorized as slow (<50% signal increase), medium (50–100%), or rapid (>100%). Meanwhile, delayed-phase enhancement was classified as persistent (>10% increase), plateau (no further increase), or washout (>10% signal decrease after the initial rise).

Inter-reader agreement was assessed between the two breast radiologists before consensus. Cohen’s kappa coefficients were calculated for BI-RADS internal enhancement patterns, the presence of mixed IEP, cystic structures on STIR images, early- and delayed-phase kinetic curve types, and diffusion restriction on DWI.

To reduce the impact of missing data, only patients with complete and diagnostically evaluable MRI and histopathology records were included in the final analysis. Eight patients were excluded: two were lost to follow-up after imaging and did not undergo biopsy, and six had MRI studies that were either missing essential sequences or rendered non-diagnostic due to motion or technical artifacts.

### 2.3. Statistical Analysis

Given the retrospective design and the relative rarity of segmental NME, no a priori sample size calculation was performed. Instead, we included all consecutive eligible cases that met the imaging and histopathologic criteria during the study period. After data collection, a post hoc power analysis (α = 0.05, power = 0.80) showed that our final sample (*n* = 103; 70 malignant, 33 benign) provided 80% power to detect a medium effect size (Cohen’s d = 0.60), whereas smaller effects may have gone undetected.

Data analysis was conducted using the Statistical Package for the Social Sciences software version 23.0, and a *p*-value < 0.05 indicated a statistically significant difference. Descriptive statistics were used to summarize the demographic and lesion characteristics. The Pearson’s chi-square test and Fisher’s exact test were utilized to assess associations between variables. The associations between the imaging findings and histopathological outcomes were explored via univariate analyses. Univariate comparisons were exploratory and intended for initial variable screening; therefore, *p*-values were not adjusted for multiple comparisons. Variables with a *p* value < 0.10 in univariate tests were entered into a multivariate logistic regression model to calculate odds ratios (ORs). Variable selection was performed using the backward stepwise method, and model fit was assessed with the Hosmer–Lemeshow test. Multicollinearity was assessed using tolerance and variance inflation factor (VIF) values (tolerance range: 0.58–0.89; VIF range: 1.12–1.71); all variables showed acceptable collinearity.

Cut-off values for enhancement patterns and kinetic parameters were determined according to previously reported thresholds in the literature and those commonly used in clinical practice; no additional ROC-based (e.g., Youden index) optimization was performed.

## 3. Results

A total of 103 patients with segmental NME lesions were included in the final analysis. The findings are presented according to patient and histopathological characteristics, internal enhancement patterns, multiparametric MRI features, and logistic regression outcomes. Detailed results are summarized below, with tables and figures illustrating the key observations.

### 3.1. Patient and Histopathological Characteristics

We examined 103 segmental NME lesions in 103 patients, with an average age of 44.38 ± 9.91 (range: 22–75) years. Of them, 70 (68%) presented with malignant lesions and 33 (32%) with benign lesions. The patients with malignant lesions were aged 26–75 (mean: 45.2) years. Among the 70 malignant tumors, 61 were ductal, 5 lobular, and 4 papillary. Approximately 40% of these malignant lesions were invasive, and 60% in situ. Seven patients had both invasive and in situ components. These lesions were categorized as invasive. Figure 1 depicts the histopathological diagnostic results.

### 3.2. Multiparametric Breast MRI Findings

#### 3.2.1. Internal Enhancement Patterns

**Figure 2:**
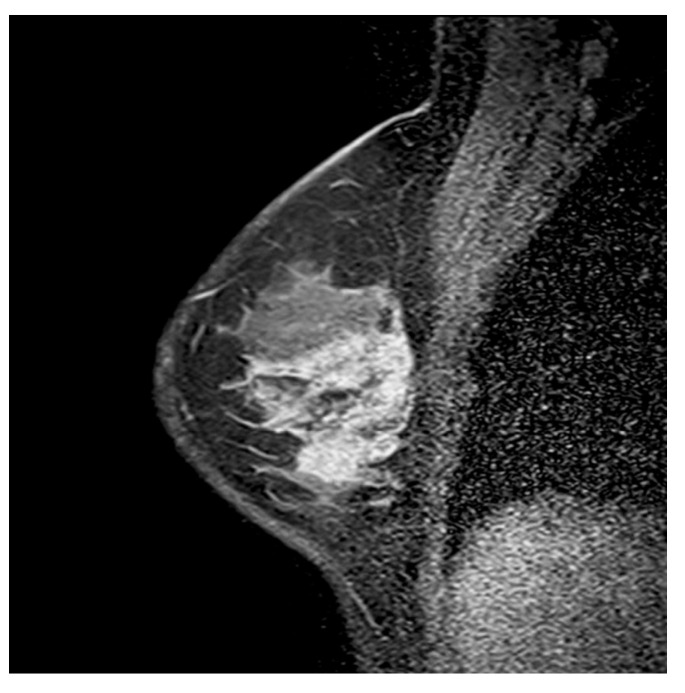
Sagittal fat-suppressed post-contrast T1-weighted breast MRI of a 46-year-old woman demonstrates segmental non-mass enhancement in the lower outer quadrant of the right breast. The lesion shows a clumped and CRE internal enhancement pattern without an associated mass, and histopathological evaluation confirmed ductal carcinoma in situ.

In our study group, heterogeneous IEPs were observed in 32% of the lesions, CRE in 31.1%, clumped patterns in 29.1%, and homogeneous patterns in 7.8%. Based on the Pearson chi-square test, there was a statistically significant correlation between IEP and histopathological outcomes (*p* = 0.002). CRE was found in 40% of malignant segmental NME lesions, and a significant correlation was observed (*p* = 0.004). CRE exhibited a sensitivity of 40%, specificity of 88%, and PPV of 87.5% for diagnosing malignancy, all with a 95% confidence interval (CI). Heterogeneous patterns were most typically noted in benign lesions, with a statistically significant difference (OR = 0.227, *p* = 0.001). Based on the univariate logistic regression (ULR) analysis, CRE increased the likelihood of malignancy by 4.8-fold (OR = 4.833, *p* = 0.007). Clumped and homogeneous patterns were not statistically significant (Table 1). An illustrative example of segmental NME in the lower outer quadrant of the breast, showing a predominantly clumped internal enhancement pattern with focal clustered ring components and histologically confirmed ductal carcinoma in situ, is presented in Figure 2.

#### 3.2.2. Mixed Enhancement Patterns

**Figure 3:**
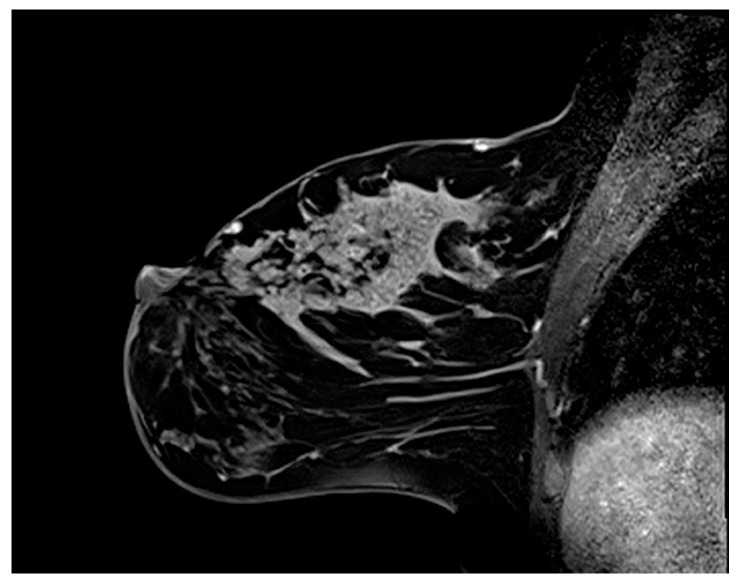
Breast magnetic resonance imaging of a 36-year-old woman. Sagittal fat-suppressed post-contrast T1-weighted image shows segmental nonmass enhancement with a heterogeneous internal enhancement pattern. Both clumped and clustered ring enhancement features are observed. Histopathological examination confirmed the coexistence of invasive ductal carcinoma and ductal carcinoma in situ.

Of the 75 lesions in which mixed IEP could be assessed (Figure 3), 26 (34.7%) presented with a mixed enhancement pattern. Approximately 41.55% of the patients with malignant lesions had mixed IEP. Of them, 15.4% exhibited benign lesions and 84.6% malignant lesions. The association between mixed IEP and malignancy did not reach conventional statistical significance (*p* = 0.053). In ULR analysis, the presence of mixed IEP was associated with a 3.19-fold increased likelihood of malignancy (OR = 3.19, *p* = 0.061).

#### 3.2.3. STIR and Diffusion MRI Findings

**Figure 4:**
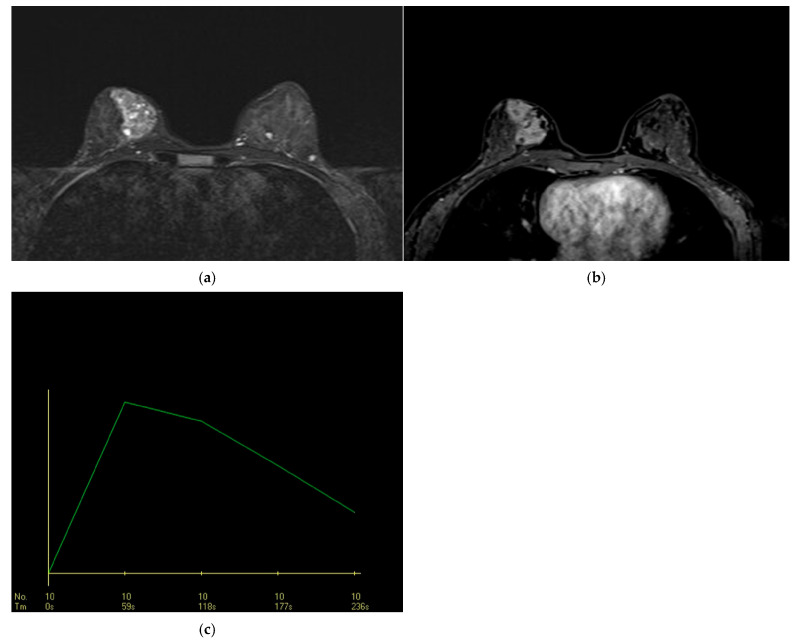
Breast MRI of a 37-year-old woman with segmental non-mass enhancement (NME). (**a**) Axial STIR sequence shows hyperintense foci within the segmental NME lesion, consistent with small cystic components. (**b**) Axial fat-suppressed post-contrast T1-weighted image shows segmental NME with a mixed internal enhancement pattern, combining clustered-ring and heterogeneous components. (**c**) DCE time–intensity curve shows rapid initial enhancement with delayed washout. Histopathology: ductal carcinoma in situ.

Cystic structures on STIR sequences were observed in 27.2% of lesions, 30.7% of which were benign and 69.3% were malignant (Figure 4). The frequency rate of cystic structures in the malignant lesions was 25.7%, which was not statistically significant (*p* = 0.625). Diffusion restriction was found in 60.9% of lesions (64.1% in malignant lesions and 57.6% in benign lesions), without statistical significance (*p* = 0.533). The ULR analysis showed a 1.3-fold higher likelihood of malignancy in lesions with diffusion restriction (OR = 1.31, *p* = 0.534); however, this result was not significant.

#### 3.2.4. DCE-MRI Kinetic Features

In the DCE-MRI assessment, the most common dynamic curve pattern was rapid contrast enhancement in the initial phase (46.6%), with a plateau pattern in the delayed phase (45.6%). Rapid early-phase contrast uptake was observed in 60% of malignant lesions, with a significantly strong correlation (*p* < 0.001). It had a sensitivity of 60%, specificity of 82%, and PPV of 87.5% (95% CI, 76.8–98.2). Further, based on the ULR analysis, rapid contrast uptake was associated with a 6.75-fold increase in the likelihood of malignancy, which was statistically significant (OR: 6.750, *p* < 0.001) (Figure 4).

The most frequent dynamic curve pattern during the delayed phase was a plateau, which was observed in 44.3% of malignant lesions and 48.5% of benign lesions, without a significant difference (*p* = 0.690). The washout pattern was the second most common, which was found in 31.4% of malignant lesions and 6.1% of benign lesions, with a strong association with malignancy (*p* = 0.004). The washout curve had a sensitivity of 31%, specificity of 94% (95% CI, 79.8–99.3), and PPV of 91% (95% CI, 76.5–97.8). Further, according to the ULR analysis, it increased the likelihood of malignancy by 7.1-fold, which was statistically significant (OR: 7.104, *p* = 0.011).

In DCE-MRI, a slow initial-phase contrast uptake was observed in 60.6% of benign lesions. This finding was significantly different from that in malignant lesions (*p* < 0.001). Approximately 45.5% of benign lesions presented with persistent enhancement curves in the delayed phase, which was also statistically significant (*p* = 0.030). According to the ULR analysis, the ORs were 0.134 for slow contrast uptake (*p* < 0.001) and 0.385 for persistent enhancement in the delayed phase (*p* = 0.033), both of which were statistically significant.

#### 3.2.5. Inter-Reader Agreement and Consensus Reading

Inter-reader agreement between the two breast radiologists was substantial to almost perfect for all imaging descriptors. Kappa values were 0.95 for BI-RADS internal enhancement patterns, 0.94 for mixed IEP (assessed in 75 lesions), 0.92 for cystic structures on STIR, 0.91–0.94 for early- and delayed-phase kinetic curves, and 0.77 for diffusion restriction on DWI.

### 3.3. Multivariate Analysis

A comprehensive multivariate logistic regression analysis was performed. In this analysis, variables with *p* < 0.10 on univariate analysis, including CRE, heterogeneous enhancement, initial-phase kinetic curves (slow and rapid), delayed-phase patterns (persistent and washout), and mixed IEP, were incorporated. In the multivariate analysis, only rapid and slow initial-phase enhancements remained statistically significant. Rapid initial-phase enhancement increased the likelihood of malignancy by 5.133-fold (*p* = 0.031). Meanwhile, slow enhancement was associated with benignity (OR = 0.194, *p* = 0.020). In the univariate analysis, CRE increased the likelihood of malignancy by 4.833-fold and the washout-type delayed enhancement by 7.104-fold, which were both statistically significant. These findings underscore the diagnostic value of early-phase kinetics and specific enhancement patterns in differentiating malignant segmental NME lesions. Table 2 presents the results of the univariate and multivariate regression analyses.

### 3.4. Correlation with Invasion

In the malignant subgroup (*n* = 70; 42 in situ, 28 invasive), we compared all multiparametric MRI features between in situ and invasive cancers. The distribution of individual BI-RADS internal enhancement patterns, the presence of cystic structures on STIR, diffusion restriction on DWI, and early- or delayed-phase kinetic curve types did not differ significantly between groups (all *p* > 0.05). Mixed IEP was more frequent in invasive than in in situ lesions (59.1% vs. 29.0%; χ^2^ *p* = 0.029, continuity-corrected *p* = 0.057), but his difference did not reach conventional statistical significance. Overall, in this cohort, multiparametric MRI did not provide robust imaging markers for reliably distinguishing in situ from invasive segmental NME.

## 4. Discussion

NME lesions with segmental distribution were evaluated according to the ACR BI-RADS 5th edition lexicon [1]. CRE was the second most common pattern after heterogeneous enhancement and the most frequent IEP in malignant lesions, with statistical significance (*p* < 0.05). The PPV of CRE was 87.5% (95% CI, 76.8–98.2), which is consistent with the literature. Its specificity was 88% (95% CI, 74.2–94.4), which is higher than the values reported in the literature. Consistent with prior reports, the diagnostic contribution of diffusion restriction, T2/STIR hyperintensity, and kinetic curves remains variable; pooled analyses indicate lower accuracy for DCE alone and improved performance for DCE + DWI, yet no single modality or descriptor is definitive [6,7]. This discrepancy might be attributed to the fact that our study focused solely on segmental NME lesions with the highest PPV. Previous studies have clearly reported a strong association between segmental NME and DCIS [3,4,11,12,13,18]. Table 3 summarizes previously reported histopathologic diagnoses associated with segmental NME lesions for contextual reference. Overall, this multicenter, distribution-stratified cohort provides lesion-specific, multiparametric evidence for segmental NME. These findings may inform the refinement of diagnostic algorithms and reporting practices. In segmental NME, our findings suggest that the presence of clustered-ring enhancement and/or rapid early-phase enhancement—particularly with delayed washout—should prompt consideration of a BI-RADS upgrade and tissue sampling, whereas slow early-phase with persistent delayed-phase kinetics in the absence of CRE may support short-interval MRI surveillance in carefully selected, clinicoradiologically concordant cases.

Heterogeneous IEP was the most common pattern in the cohort overall, driven by its prevalence in benign lesions (18/33, 54.5%). In malignant lesions, the least frequent IEP was homogeneous (7/70, 10.0%), whereas heterogeneous accounted for 15/70 (21.4%). Heterogeneous enhancement showed a significant inverse association with malignancy (OR = 0.227, *p* = 0.001), while clustered-ring enhancement predominated among malignant cases (28/70, 40%; *p* = 0.004). These findings are consistent with prior reports noting variable links between heterogeneity and malignancy [10].

Homogeneous IEP was the least observed pattern and was not significantly associated with malignancy. The prevalence rate of malignancy in segmental NME lesions with a clumped contrast pattern was 66.7%. However, its association with malignancy was not statistically significant (*p* > 0.05), which is consistent with previous studies [4,9].

Mixed IEP, where two or more IEP types were present within the same lesion, was observed in 34.7% of cases. Among these, 84.6% were malignant lesions. In our cohort, mixed IEP showed a borderline association with malignancy (OR = 3.19, *p* = 0.061). Although these findings did not reach conventional statistical significance, the effect size suggests that larger, adequately powered series may be needed to clarify the true predictive value of mixed enhancement patterns in segmental NME. Shimauchi et al. showed that clumped and CRE patterns might have similar developmental mechanisms and occur concomitantly [13]. Our study did not specify which contrast patterns coexisted within the mixed contrast findings. However, if some cases of mixed IEP represent a combination of CRE and clumped enhancement patterns, this may partially account for the borderline statistical association observed.

Li Yan et al. showed that the diagnostic performance of DWI was inferior to that of DCE-MRI, possibly due to DWI slice thickness [16]. Our study, which focused on segmental NME, did not encounter this limitation as all lesions were larger than the DWI slice thickness. Diffusion restriction was observed in both benign and malignant lesions, with no significant correlation to malignancy. Previous studies have shown that, unlike mass lesions, the diagnostic accuracy of diffusion restriction in NME lesions is limited [14,16]. The alignment of our findings with previously published reports, within a cohort with a high malignancy risk in segmental NMEs, underscores the diagnostic limitations of diffusion restriction in NME lesions.

Baltzer et al. did not find statistically significant differences in the dynamic enhancement features between benign and malignant lesions [15]. Conversely, Liu et al. showed that the washout curves were more frequent in malignant lesions. Meanwhile, persistent curves were more common in benign ones, with statistical significance [3]. Our study identified a strong association between malignancy and rapid contrast uptake in the initial phase and washout dynamic curves in the delayed phase (*p* < 0.001 and *p* = 0.009, respectively). The literature has reported that plateau curves were more frequently observed in malignant NME lesions, unlike our findings [3,4,8,16]. In addition, Liu et al. found that washout-type curves might help distinguish invasive breast cancer from benign lesions. However, our study did not find a statistically significant correlation between the invasion and dynamic curves.

There was no statistically significant correlation between the cystic structures observed in the STIR images and the presence of benign or malignant lesions. Previous studies have contrasting findings on this matter. Milosevic et al. showed an association between the presence of microcysts on STIR and atypical lesions [23]. Baltzer and colleagues have reported that hyperintensity accompanying NME lesions on T2-weighted images is more significantly associated with benignity and hypointensity than with malignancy [15]. Conversely, Chikarmane et al. showed that hyperintensity on T2-weighted sequences is unreliable for assuming benignity in NME lesions [2].

The efficacy of MRI in distinguishing invasive NME lesions varies across studies. Liu et al. found no association between invasion and IEP. Meanwhile, Hahn et al. reported that CRE was more frequently found in microinvasive ductal carcinomas than in DCIS [3,24]. Machida et al. found a significant correlation between CRE and malignancy, indicating that IEPs could predict invasion, with PPV values of 40.2% for clumped patterns, 46.2% for branching, 51.8% for heterogeneous enhancement, 50% for CRE, and 73.9% for hypointense areas, which are not included in the MRI BI-RADS atlas [12]. Using the ACR BI-RADS 5th edition criteria, our study did not find significant predictors of invasion, possibly due to the exclusion of specific descriptors analyzed in a study of Machida et al.

Future studies should employ prospective, protocol-standardized multicenter cohorts with harmonized DCE temporal resolution (≤10–15 s per phase), field strength and coil specifications, DWI b-values (≥800 s/mm^2^) and ADC mapping, and uniform ROI rules and reporting aligned to the BI-RADS lexicon; such standardization would reduce technical variability and facilitate external validation.

This study has several limitations. First, its retrospective design might have introduced selection bias and resulted in incomplete imaging and clinical records despite the predefined criteria. In particular, the non-random, referral-based nature of clinical breast MRI and the exclusion of non-diagnostic examinations may have enriched the prevalence of malignancy in our cohort and may limit the generalizability of the estimated predictive values to screening or lower-risk populations. Second, the multicenter setting, with varying MRI field strengths and imaging parameters, might have led to inconsistencies in image resolution and IEP evaluation, particularly in differentiating CRE from clumped patterns. Although the acquired sequences were similar, the parameter variations across centers remain a limitation. However, the multicenter approach improves patient diversity and enhances generalizability. Missing data were handled using a complete-case approach (no statistical imputation) under an MCAR assumption; although exclusions were few (*n* = 8; two lost to follow-up and six non-diagnostic MRI), any departure from MCAR could introduce bias and limit generalizability. Moreover, univariate *p*-values were not adjusted for multiple comparisons and should be interpreted as exploratory/descriptive. Observer bias was decreased by having experienced radiologists independently review the images. However, interobserver variability remains a potential limitation. Finally, a post hoc power analysis (α = 0.05, power = 0.80) showed that our sample (*n* = 103; 70 malignant, 33 benign) had 80% power to detect a medium effect (Cohen’s d = 0.60). Smaller effects may have gone undetected, supporting the need for larger cohorts to improve precision. In interpreting our findings, the primary inferential emphasis was placed on the multivariate logistic regression results; univariate *p*-values, particularly those close to the 0.05 threshold, should be regarded as exploratory/descriptive in the context of multiple testing.

## 5. Conclusions

In conclusion, our study offers a comprehensive examination of segmental NME lesions and their multiparametric MRI findings in relation to malignancy and invasion across a broad patient population. The results can provide significant insights into distinguishing benign from malignant lesions and identifying the presence of invasive components. Further, they can contribute significantly to the current body of literature and clinical scenarios encountered in daily practice, thereby setting a foundation for future multicentric and extensive studies to better understand less common NME patterns.

## Figures and Tables

**Table 1:** MRI characteristics of segmental NME lesions.

Characteristics	Benign Lesions (*n* = 33, 32%) *n* (%)	Malignant Lesions (*n* = 70, 68%) *n* (%)	*p* Value
Internal enhancement pattern *			0.002 *
Clustered ring *	4 (12.1%)	28 (40%)	0.004 *
Clumped	10 (30.3%)	20 (28.6%)	0.857
Heterogeneous *	18 (54.6%)	15 (21.4%)	0.001 *
Homogeneous	1 (3.0%)	7 (10.0%)	0.431 **
Mixed enhancement pattern (*n* = 75)			
Present	4 (18.2%)	22 (41.5%)	0.053
Absent	18 (81.8%)	31 (58.5%)	
Dynamic curve in the initial phase *			<0.001 *
Slow *	20 (60.6%)	12 (17.1%)	<0.001 *
Moderate	7 (21.2%)	16 (22.9%)	0.852
Rapid *	6 (18.2%)	42 (60%)	<0.001 *
Dynamic curve in the delayed phase *			0.009 *
Persistent	15 (45.5%)	17 (24.3%)	0.030 *
Plateau	16 (48.5%)	31 (44.3%)	0.690
Washout	2 (6.0%)	22 (31.4%)	0.004 *
Diffusion restriction			
Present	19 (57.6%)	41 (64.1%)	0.533
Absent	14 (42.4%)	23 (35.9%)	
Cystic structures on the STIR images			
Present	10 (30.3%)	18 (25.7%)	0.625
Absent	23 (69.7%)	52 (74.3%)	

*: *p* < 0.050 **: Fisher’s exact test.

**Table 2:** Results of the univariate and multivariate logistic regression analyses.

	Univariate Analysis				Multivariate Analysis			
	OR	95% CI for EXP(B)		*p* Value	OR	95% CI for EXP(B)		*p* Value
		Lower	Upper			Lower	Upper	
Internal enhancement pattern								
Clustered ring *	4.833	1.531	15.258	0.007 *				
Clumped	0.920	0.372	2.275	0.857				
Heterogeneous *	0.227	0.093	0.554	0.001 *				
Homogeneous	3.556	0.419	30.161	0.245				
Dynamic curve in the initial phase								
Slow *	0.134	0.053	0.343	0.000 *	0.194	0.049	0.770	0.020
Moderate	1.101	0.403	3.003	0.852				
Rapid *	6.750	2.469	18.451	0.000 *	5.133	1.164	22.637	0.031
Dynamic curve in the delayed phase								
Persistent *	0.385	0.160	0.925	0.033 *				
Plateau	0.845	0.368	1.936	0.690				
Washout *	7.104	1.559	32.363	0.011 *				
Mixed IEP	3.194	0.949	10.746	0.061				
Diffusion restriction	1.314	0.557	3.100	0.534				

*: *p* < 0.050.

**Table 3:** Histopathological diagnoses associated with NME lesions reported in the literature [2,3,4,18,19,20,21,22].

Classification	Lesions
Benign	Pseudoangiomatous stromal hyperplasia, usual ductal hyperplasia, apocrine metaplasia, adenosis and fibrocystic changes, duct ectasia and periductal fibrosis, fibroadenoma, silicone granuloma, sclerosing adenosis, inflammation, granulomatous mastitis, post-radiation changes, normal breast tissue
High-risk	Atypical ductal hyperplasia, intraductal papilloma, peripheral papillomatosis, radial scar, complex sclerosing lesion, flat epithelial atypia
Malignant	Ductal carcinoma in situ (DCIS), invasive ductal carcinoma, invasive lobular carcinoma, tubular carcinoma, inflammatory breast cancer, papillary carcinoma, mucinous carcinoma, apocrine carcinoma, invasive micropapillary carcinoma, Paget’s disease, glycogen-rich clear cell carcinoma

## Data Availability

The data presented in this study are available on request from the corresponding author. The data are not publicly available due to privacy and ethical restrictions.

## Abbreviations

The following abbreviations are used in this manuscript:

ACR	American College of Radiology
BI-RADS	Breast Imaging Reporting and Data System
CRE	clustered ring enhancement
DCE	dynamic contrast-enhanced
DCIS	ductal carcinoma in situ
DWI	diffusion-weighted imaging
IDC	invasive ductal carcinoma
IEP	internal enhancement pattern
MRI	magnetic resonance imaging
NME	nonmass enhancement
OR	odds ratio
PPV	positive predictive value
STIR	short tau inversion recovery
TIC	time–intensity curve
